# Prevalence and factors associated with stunting and thinness among school age children in rural primary schools, East Dembia District, Northwest Ethiopia

**DOI:** 10.1186/s40795-022-00624-6

**Published:** 2022-11-09

**Authors:** Mihretu Sisay, Azeb Atenafu, Melkamu Tamir Hunegnaw, Merkineh Markos Lorato

**Affiliations:** 1 World Vision Ethiopia Dembia Area Program, Gondar, Ethiopia; 2 Department of Human Nutrition, Institute of Public Health, College of Medicine and Health Science, University of Gondar, Gondar, Ethiopia; 3 School of Public Health, College of Health Science, Woldia University, Woldia, Ethiopia

**Keywords:** Stunting, Thinness, Under-nutrition, School children, Dembia, Ethiopia

## Abstract

**Purpose:**

To assess the prevalence and factors associated with stunting and thinness among school-age children in rural primary schools in the East Dembia District, Northwest Ethiopia.

**Methods:**

An institution-based cross-sectional study was conducted using a systematic random sampling procedure to select 840 school-aged children. A structured interviewer-administered questionnaire was used to collect the data. Height and weight measurements were taken, and a combined wet mount and concentration technique was used. Epi Data 3.1 was used to enter data, which was then exported to SPSS version 20 for analysis. Bi-variable and multivariable logistic regression analyses were done. Variables with a *p*-value of less than 0.05 were considered significantly associated with stunting and thinness.

**Results:**

The prevalence of stunting and thinness was 25.5 and 13.0%, respectively. Being infected with an intestinal parasite (AOR =4.34; 95% CI: 2.52, 12.27), being in the age group 11–14 years (AOR =3.73; 95% CI: 2.19, 6.34), having the lowest dietary diversity practice (AOR =4.61; 95% CI: 1.73, 12.27), unimproved water sources (AOR =1.76; 95% CI: 1.07, 2.91), not practicing good hygiene practice (AOR =1.71; 95% CI: 1.04, 2.804) and having an unimproved latrine type (AOR =1.72; 95% CI: 1.03, 2.89) were significantly associated with stunting. On the other hand, unsecured food (AOR =1.74; 95% CI: 1.08, 2.81), eating less than 3 meals per day (AOR = 2.67; 95% CI: 1.11, 6.46), and untreated water (AOR =1.72; 95% CI: 1.08, 2.75) were factors associated significantly with thinness.

**Conclusion:**

Stunting and thinness are predominant public health problems in the study area, provided that the prevalence of stunting is slightly higher than that of a national survey on health and nutrition in schoolchildren, whereas the prevalence of thinness is lower when compared to the same national survey. In this study, the primary factor that was significantly associated with stunting was dietary diversity; the primary factor that was associated with thinness was eating fewer than 3 meals per day. So, an integrated strategy is important to alleviate undernutrition among school-aged children in the current study area.

## Introduction

Malnutrition refers to either a deficiency or excess in the intake of calories, proteins, and/or other nutrients. It is a major public health problem in the world, leading to child morbidity, particularly in developing countries [1, 2].

For children, school-age is the age of changes like the development of self-efficacy; the knowledge of what to do and the ability to do it can be highly structured [3, 4]. Unfortunately, these children are vulnerable to nutritional deficiencies because of the greatest demand for nutrients [3]. Good nutrition is an essential requirement for meeting the increased biological demand and maintaining school-aged children’s health [5, 6].

Age [7], food insecurity of households [8], educational status, and occupation of parents [9, 10] were some of the factors that were significantly associated with under-nutrition in previous studies.

Under-nutrition in children results in low cognitive development and poor school performance, increases school dropout and repeated absenteeism, exposes them to infection and increases health care expenditure, reducing human capital. It contributes to 21% of disability-adjusted life-years for children, and particularly stunting, is responsible for the loss of 2–3% of gross domestic product (GDP) in the country [11].

Stunting is estimated to affect 48–56% of school-aged children worldwide [12]. The national school health and nutritional survey in Ethiopia showed that the prevalence of stunting and thinness was 22.1 and 23.3%, respectively [13]. In the same study, the second largest prevalence of stunting was seen in Amhara regional state (32.1%). Thus, assessing the nutritional status of schoolchildren is important [14]. The nutritional status of children is also used to assess the quality of their living environments [15, 16].

In addition, despite the implementation of school health and nutritional programs, the problem of malnutrition is still not properly addressed [17]. Hence, to the best of our knowledge, under-nutrition affects children living in rural areas disproportionately as compared to their urban counterparts [18]. In addition, little attention is given to school-aged children in comparison to under-five children [19], which hides the prevalence and significant factors of under-nutrition. Therefore, the aim of this study was to assess the prevalence of stunting and thinness and associated factors among school-age children in rural primary schools in the East Dembia District, Northwest Ethiopia.

## Methods

### Study area and design

The study was conducted in rural primary schools in the East Dembia district, Northwest Ethiopia. The district is located 745 km away from Addis Ababa, the capital city of Ethiopia. The district has 34 Kebele, which is the smallest administrative unit (4 urban Kebele and 30 rural Kebele). According to the 2019 Fiscal Year population projection report, the total population of the district is estimated to be 202,534. Of which, the number of school-aged children is estimated to be 60,262 (29,529 boys and 30,733 girls).

The district had 56, 28 and 3 elementary, primary, and secondary schools, respectively. Overall, a total of 31,682 children were registered in the schools. Among the total registered primary school children, 22,907 students (11,914 boys and 10,993 girls) were found in the rural kebele primary schools [20].

An institution-based cross-sectional study design was applied to assess the prevalence and factors associated with stunting and thinness among school-age children in rural primary schools of East Dembia District, Amhara Regional State, Ethiopia, from 1 February to 5 March, 2020.

The source population consisted of all children in the age group between 6 and 14 years old who were enrolled in East Dembia district primary schools.

The study population was all children whose age was 6 to 14 years old among the selected rural primary schools in the East Dembia district. All children who were aged 6 to 14 years old from rural primary schools and who attended the school during the data collection period were included, whereas seriously ill children and those unable to stand upright by themselves were excluded from the study.

### Sample size determination and sampling technique

The sample size was determined by using the single population proportion formula by taking the prevalence of under-nutrition as 46.1% from the study conducted at Gondar, Ethiopia [21], the Z statistic of 1.96, margin error of 5%, and non-response rate of 10%, with the consideration of a design effect of 2. Then, the calculated total sample size was *840*.

A systematic random sampling procedure was employed to select a sample of school-age children from the rural primary schools. In the first stage, by using a simple random sampling technique, eight primary schools were selected from 26 full-cycle (grade 1–8) rural primary schools. Then, a proportional allocation was made to selected schools based on the number of pupils in the respective school. Finally, using the student roster as a sampling frame, a systematic random sampling technique was employed to select every eleventh child [by dividing the total number of students in eight selected schools (i.e., 8958) to the calculated sample size (840). The random starting was seven, obtained by using the lottery method.

### Dependent variables

Under-nutrition (Stunting and Thinness).

### Independent variables

Socio-demographic and economic factors include: sex, age of the child, marital status, educational status, employment status, religion**,** family size, and wealth index.

Health conditions**:** sickness, types of illness, presence of intestinal infectious diseases.

Dietary diversity and feeding practices: dietary diversity in the home, meal frequency per day.

Food Security: Household Food Insecurity.

Water, sanitation, and hygiene factors: availability of clean water, safe sanitation facilities, and hygienic practice.

### Operational definitions

“School-age children”: a child between the ages of 6 and 14 years old [22].

Under-nutrition: children with any of the two forms of malnutrition (stunting or wasting).

Stunting: height for age < − 2 SD of the WHO Child Growth Standards median.

Thinness: BMI for age < − 2 SD from the WHO Child Growth Standards median [23].

Access to clean water: “the proportion of the population using any piped water, public tap, protected spring or well” [24].

Improved sanitation facility: an excreta disposal facility with no feces on the floor and properly constructed [25].

Socioeconomic status was measured using a tercile index derived from household assets and utilities scores, and the wealth tercile was divided into rich, middle, and poor.

Household food secure: A household is considered food secure when the summation is less than 2 of 27 food insecurity indicators.

#### Dietary diversity

The pattern of dietary diversity is classified as lowest (< 4), medium (5–7) and highest (> 8) [26].

### Data collection tools and procedure

Data was collected using a structured questionnaire via a face-to-face exit interview. The child guardian was traced at the household level for wealth index, household food insecurity, and dietary diversity pattern. The questionnaire was adapted from different literature [2, 5, 8, 18, 27] and guidelines [28, 29]. Additionally, an anthropometric measurement and laboratory investigation for the stool sample were included in the data collection tools. Socio-demographic and economic characteristics; water, hygiene, and sanitation characteristics; household dietary diversity by using 24 hour recall methods; and the household food insecurity access scale, which was developed by the Food and Nutritional Technical Assistance (FANTA) [30–32], were included in the questionnaire. The household Dietary Diversity Score (DDS) was categorized as low when the DDS was below 4, medium when the DDS was 5 to 7, and high when the DDS was greater than 8 [31].

Age, weight, and height were used to determine z scores for height-for-age (HAZ) and BMI-for-age (BAZ) or thinness. The weight of study subjects was measured with minimum clothing and no shoes to the nearest 0.1 kg. The weight scale was digital and calibrated by 2 kg iron bar every day. Height was measured by using a standiometer in a standing position to the nearest 0.1 cm. When the data collectors took the height of the study subject, the following points were checked: heel, buttocks, shoulder touching the vertical stand of the stadiometer, and adjusting of the head to be perpendicular to the Frankfurt plane was checked. Children with HAZ and BAZ < -2 SD were classified as stunted and thin, respectively. Undernourished children were those who had at least one form of under-nutrition index [23].

Each student was given a clean, labeled stool cup as well as an applicator stick to collect approximately 2 g of a fresh stool sample. The collected stool sample was emulsified with 0.9% saline and 10% formalin solution to maintain the quality of the sample [33] and examined using wet mount and concentration techniques combined. For this purpose and data collection, two medical laboratory technicians and one laboratory technologist were assigned.

### Data quality control

The data collection tool had been prepared in English and translated into the local Amharic language by language experts. Then, back to the English language to check for consistency. Before the actual survey was started, one-day training was given for data collectors and supervisors on how to approach and collect the data. A pre-test was done on 42 (5%) of the sample size at a primary school in Gondar, Zuria woreda before the actual data collection began. The collected data was checked for completeness, accuracy, and clarity by the supervisor and principal investigator. The quality of the stool laboratory investigation was maintained using standard operating procedure. Before conducting the analysis, the data was cleaned and cross-checked.

### Data processing and analysis

Version 4.6.0.2 of Epi-Data was used to enter data. Then, it was exported and analyzed using SPSS version 20 software. Age, weight, and height have been exported from Epi-Data software and imported to AnthroPlus software version1.4.1 to calculate height for age Z scores (HAZ) and BMI for age Z scores (BAZ) [34]. The household wealth index was computed by considering the household assets (i.e. livestock, type of house, durable assets, productive assets, and agricultural land ownership).

Regression is used to evaluate the association between variables, and descriptive tests state prevalence, mean, percent, and frequency are performed to express the results of this study.

A bi-variable analysis was performed, and variables that had a *p*-value of less than 0.2 were selected for multivariable analysis. Finally, multivariable analysis was performed, and variables with a *p*-value less than 0.05 were considered statistically significant. Model fitness was also checked by using the Hosmer-Lemeshow test.

### Ethical consideration

An ethical clearance was obtained from the ethical review board of the University of Gondar and the study has been performed in accordance with the ethical standards laid down in the 1964 declaration of Helsinki and its advanced revisions. Then, a letter of permission was gained from the East Dembia district Education office. Written informed consent was obtained from parents or guardians prior to the inclusion of the study subjects, while informed assent was taken from children to participate in the study. Details like names of participants and identification numbers that might disclose the identity of participants were not included in the data collection tool. The purpose of the study was briefly explained to the participants and guardians before the interview. The right to withdraw from the study was given to the study participants whenever they wanted to do so. During the course of data collection, an anti-helminthes drug was given for those children who had positive results for intestinal parasite infection.

## Results

### Socio-demographic and economic characteristics

In this study, a total of 821 school-aged children participated with a response rate of 97.7%. The mean age of respondents was 11.11 (SD ± 4.85) years. More than half (425 or 51.8%) of the study participants were male. Ninety nine point 1 % (99.1%) of those who took part were orthodox Christian believers. In the current study, 677 (82.5%) of mothers were unable to read and write, while more than two-thirds 570(69.5%) of fathers had not attended formal education. In this study, (91.9%) and (92.4%) of the participants’ parents’ or guardians were housewives and farmers, respectively. Nearly three-fourths (78.6%) of the households had a family size of 5–7 (Table 1).

**Table 1:** Socio-demographic and economic characteristics of school age children in Rural Primary Schools of East Dembia District, Northwest Ethiopia (*n* = 821), 2020

Variable	Category	Frequency	Percent (%)
Sex	Male	396	48.2
	Female	425	51.8
Student Age	6–10	305	37.1
	11–14	516	62.9
Religion	Orthodox Christian	814	99.1
	Others^a^	7	0.9
Mother educational status	Not formal education	677	82.5
	Primary education	125	15.2
	Secondary and above	13	1.6
Father educational status	Not formal education	570	69.4
	Primary education	198	24.1
	Secondary and above	32	3.9
Mothers’ occupational status	Housewife	771	93.9
	Daily labor	12	1.5
	Merchant and Gov’t employee	31	3.8
Fathers’ Occupational status	Farmer	735	89.5
	Daily labor	27	3.3
	Merchant and Gov’t employee	31	3.8
Family size	< 4	71	8.6
	5–7	654	79.7
	> 8	96	11.7
Wealth status	Poor	273	33.3
	Medium	242	29.4
	Rich	306	37.3

Descriptive statistics; ^a^Muslims, Protestant Christian, and catholic

### Household dietary diversity and food insecurity

Only 8.9% of the households had good dietary diversity practices. More than half (52.4%) of the households used animal products in the past 24 h. More than three-fourths (76.3%) of the school-age children consumed meals three times per day. About 83.6% of children had eaten breakfast regularly before they came to school (Table 2).

**Table 2:** Household dietary diversity practice and food security of school age children in rural primary schools of East Dembia district, Northwest Ethiopia (*n* = 821), 2020

Variables	Category	Frequency	Percent (%)
Dietary diversity	Lowest	154	18.8
	Medium	594	72.4
	Highest	73	8.9
Meal frequency per day	Two per day	47	5.7
	Three per day	627	76.4
	Four per day	147	17.9
Take breakfast before coming to school	Yes	685	83.4
	No	136	16.6
Food security	Secured	335	40.9
	unsecured	484	59.1

Descriptive statistics

### Water, sanitation, and hygiene

Households obtaining “clean water” from improved water sources were 72.1%. About 61.8% of children utilized non-treated water. Households that have had a toilet facility for the defecation of feces were estimated to be 67.2%. Only One-third (33.6%) of participants practiced good hygiene, such as trimming their nails, avoiding dirtiness from their nails, and wash their hands with soap or ash at the critical time (Table 3).

**Table 3:** Water, Sanitation and Hygiene status of school age children in rural primary schools of East Dembia district, Northwest Ethiopia (*n* = 821), 2020

Variable	Category	Frequency	Percent(%)
Hygiene Practice	Good hygiene	276	33.6
	Not good	545	66.4
Latrine Availability	In latrine	552	67.2
	Open field	269	32.8
Types of Latrine	Improved	369	66.8
	Not Improved	183	33.2
Water source	Improved	592	72.1
	Not improved	229	27.9
Water treating before using	Treated	313	38.1
	Not treated	507	61.8

Descriptive statistics

### Health condition of the child

Eighteen percent 150(18.3%) of children experienced illness during the two-week period before study was conducted. Three-fourths (67.3%) of them experienced diarrhea and intestinal parasitic disease. In stool examination, 158 (19.2%) of participants had an intestinal parasitic infection (Table 4). The most prevalent parasites were Ascariasis 13(8.2%), followed by Amebiasis 7 (4.3%).

**Table 4:** Health condition of school age children in rural primary schools of East Dembia district, Northwest Ethiopia (*n* = 821), 2020

Variable	Category	Frequency	Percent (%)
History of Illness	Yes	150	18.3
	No	671	81.7
Types of illness	Diarrheal and intestinal parasite disease	101	12.3
	Malaria	47	4.9
	Others	9	1.1
Loss of appetite due to illness	Yes	60	40
	No	90	60
Intestinal parasite infestation	Infected	158	19.2
	Not infected	663	80.8
	No	135	16.4

Descriptive statistics

### Prevalence of under-nutrition

The prevalence of stunting was 25.5% (95% CI: 22.5, 28.5) and thinness was 13.0% (95 CI: 10.7, 15.2) among study participants in rural primary schools of East Dembia district. Among the stunted, 116 (55.5%) of children were males. Eight-1 % (80.9%) of stunting was found in the age group between 11 and 14 years old. More than half of 55 (51.4%) of the thin children were males, and in the age group between 6 and 10 years, 64(59.8%).

### Factors associated with stunting and thinness

In bi-variable logistic regression, stunting was significantly associated with the variables age, sex, food security and dietary diversity practice, meal frequency, and intestinal parasite infestation, and hygiene practice, access to clean water, water treatment, and sanitation, with *p* values less than 0.2, whereas thinness was significantly associated with household food security, dietary diversity practice, age, water treatment, and meal frequency.

In multivariable logistic regression analysis, household food security, dietary diversity practice, intestinal parasite infestation, hygiene practice, age, water treatment, and latrine type were factors that showed significant association with stunting, while food security, age, water treatment, and meal frequency were significantly associated with thinness with a *p*-value less than 0.05.

Those children in the age group of 11–14 years were 3.7 times more likely to be stunted (AOR: 3.73, 95% CI: 2.19, 6.34) when compared with those aged less than 11 years. Children who practiced poor dietary diversity had a 4.6 fold chance of stunting (AOR: 4.61, 95%CI; 1.73, 12.27) compared to children who had good dietary diversity practice. The odds of stunting were 2.6 times (AOR: 2.57, 95%CI; 1.56, 4.21) higher among children who lived in food unsecured households. The odds of having stunting was 4.3 times (AOR: 4.34, 95%CI; 2.52, 7.48) higher in children who had intestinal parasite infestation than in children who were not positive for intestinal parasite infestation. Besides, children who have poor hygiene practices were 1.64 times (AOR: 1.64; 95%CI; 1.01, 2.68) more likely to be stunted as compared to children who have good hygiene practices. School children were 1.76 times (AOR = 1.76, 95% CI: 1.07, 2.91) and 1.72 times (AOR = 1.72, 95% CI: 1.03, 2.89) more likely to be stunted if their water source and latrine type were not improved (Table 5).

**Table 5:** Factors associated with stunting among school age children in rural primary schools of East Dembia district, Northwest, Ethiopia (*n* = 821), 2020

Variable	Category	Stunted		Odd Ratio	
		Stunted	Not stunted	COR (95% CI)	AOR (95% CI)
Household Food security	unsecured	163 (33.7%)	321 (66.3%/)	3.19 (2.22,4.59)	2.57 (1.56,4.21) *
	Secured	46 (13.7%)	289 (86.3%)	1	1
Dietary diversity practice	Lowest	71 (46.1%)	83 (53.9%)	6.08 (2.82,13.09)	4.61 (1.73,12.27) *
	Medium	129 (21.7%)	465 (78.3%)	1.97 (0.95,4.07)	1.51 (0.61,3.75)
	Highest	9 (12.3%)	64 (87.7%)	1	1
Intestinal parasite infestation	Infected	79 (50.0%)	79 (50.0%)	4.1 (2.84,5.91)	4.34 (2.52,7.48) *
	Not infected	130 (19.6%)	533 (80.4%)	1	1
Hygiene practice	Not Good	158 (29.0%)	387 (71.0%)	1.8 (1.26,2.57)	1.64 (1.01,2.68) *
	Good Hygiene	51 (18.5%)	225 (81.5%)	1	1
Age	6–10	40 (13.1%)	265 (86.9%)	1	1
	11–14	169 (32.8%)	347 (67.2%)	3.23 (2.2,4.72)	3.73 (2.19, 6.34) *
Water source	Improved	123 (20.8%)	469 (79.2%)	1	1
	Not improved	86 (37.6%)	143 (62.4%)	2.29 (1.64,3.2)	1.76 (1.07,2.91) *
Water treating	Treated	62 (19.8%)	251 (80.2%)	1	1
	Not treated	146 (28.8%)	361 (71.2%)	1.64 (1.16,2.29)	1.1 (0.686,1.79)
Meal frequency	Two per day	18 (38.3%)	29 (61.7%)	2.06 (1.02,4.16)	1.32 (0.48,3.61)
	Three per day	157 (25.0%)	470 (75.0%)	1.11 (0.73,1.69)	.89 (0.48,1.65)
	Four and above per day	34 (23.1%)	113 (76.9)	1	1
Latrine type	Improved	62 (19.8%)	251 (80.2%)	1	1
	Not improved	146 (28.8%)	361 (71.2%)	1.56 (1.02,2.38)	1.72 (1.03,2.89) *

Bi-variable and multi-variable logistic regression

*statistically significant at *p*-value < 0.05

*COR* Crude Odds Ratio, *AOR* Adjusted Odds Ratio, *CI* Confidence Interval

Those children who lived in food unsecured households had 1.74 times (AOR: 1.74, 95% CI; 1.08, 2.81) more likely to be thin than their counterparts. The likelihood of thinness was 2.67 times (AOR: 2.67; 95% CI; 1.11, 6.46) higher among children whose meal frequency was two or less as compared to children whose meal frequency was four or above. Also, those children who consumed non-treated water were 1.72 times (AOR: 1.72; 95% CI; 1.08, 2.75) more likely to be thin than their counterparts (Table 6).

**Table 6:** Factors associated with thinness among school age children of rural primary schools in East Dembia district, Northwest Ethiopia (*n* = 821)

Variable	Category	Thinness		Odd Ratio	
		Thin	Not thin	COR (95% CI)	AOR (95% CI)
Household Food securedd	unsecuredd	78 (16.1%)	406 (83.9%/)	2.03 (1.29,3.18)	1.74 (1.08,2.81) *
	Securedd	29 (8.7%)	306 (91.3%)	1	1
Dietary diversity practice	Lowest	34 (31.8%)	120 (16.8%)	3.16 (1.26,7.92)	2.52 (0.95,6.67)
	Medium	67 (62.6%)	527 (73.8%)	1.42 (0.59,3.38)	1.25 (0.51,3.12)
	Highest	6 (5.6%)	67 (9.4%)	1	1
Age	6–10	64 (21.0%)	241 (79.0%)	1	1
	11–14	43 (8.3%)	423 (91.7%)	0.34 (0.23,0.52)	0.31 (0.19,0.47) *
Water source	Improved	70 (11.8%)	522 (88.2%)	1	1
	Not improved	37 (16.2%)	192 (83.8%)	1.43 (0.93,2.21)	1.29 (0.82,2.04)
Water treating	Treated	29 (9.3%)	284 (90.7%)	1	1
	Not treated	78 (15.4%)	429 (84.6%)	1.78 (1.13,2.79)	1.72 (1.08,2.75) *
Meal frequency	Less than two per day	11 (23.4%)	36 (76.6%)	1.94 (0.85,4.42)	2.67 (1.11,6.46) *
	Three per day	76 (12.1%)	551 (87.9%)	0.87 (0.52,1.49)	0.89 (0.51,1.56)
	Four and above per day	20 (13.6%)	127 (86.4%)	1	1

Bi-variable and multi-variable logistic regression

*statistically significant at *p*-value < 0.05

*COR* Crude Odds Ratio, *AOR* Adjusted Odds Ratio, *CI* Confidence Interval

## Discussion

In this study, the prevalence of stunting among school-age children was 25.5% (95% CI: 22.5, 28.5). This finding is in line with the study results done in Nigeria (26%) [9], Uganda (22.5%) [10], and Debremarkos in Ethiopia (27.5%) [35]. However, the findings of this study are much lower than the study results done in Egypt (34.2%) [7], Bangladesh (55.9%) [36], and Argentina (38.9%) [37]. The possible reason may be due to the difference in maternal occupational status that may have significant effects on the provision of a balanced diet for their children. On the other hand, the current study finding was higher than the study outcome conducted in Ecuador (12.3%) [38], Mexico (18%) [39], and India (19.9%) [40]. The possible reason for the difference might be the socio-economic variation among countries.

The prevalence of thinness among study participants was 13.0%(95 CI: 10.7, 15.2), which is in line with a study conducted in Guinea Bissau (13.8%) [41], and in Burkina Faso (11.2%) [42]. However, the finding of the present study is higher as compared to the study results conducted in Nigeria (1.4%) [9], India (2%) [43], Ecuador (2.1%) [38], in southern Ethiopia (10.1%) [44] and Arbaminch (8%) [45]. Whereas, the finding of the current study is lower as compared to the study outcome done in Lalibela, Ethiopia (29.5%) [46], in the Philippines (27.8%) [47] and Uganda (18.5%) [10]. Since thinness is an acute form of undernutrition and recent weight loss, the difference may be due to the unavailability of adequate food, poor dietary diversity practice, or repeated infection.

The likelihood of stunting in children having intestinal parasitic infestation was 4.34 times when compared to their counterparts. This finding is supported by other study results conducted in Mexico [39] and Delo [48]. The possible explanation may be due to mal-absorption of nutrients “that is caused by intestinal parasites” affecting the linear growth and development of the child. Older children were 3.9 times more prone to stunting than the younger age groups. This finding is in line with the other study outcomes conducted in Egypt [7], Gondar, Ethiopia [21], and Dale, Ethiopia [48]. This may be due to a unit increment in the age of the child leading to an increment in nutritional requirements to promote the growth of a child. Thus, a lack of nutrients is responsible for stunting [49]. According to this study, children who had the lowest dietary diversity practice were 4.6 times more likely to be stunted when compared to children who had the highest dietary diversity practice. Our finding agrees with other study results carried out in Madagascar [50] and Kenya [51]. The possible reasons for similarity may be due to inadequate diet for a child’s body requirements and poor food group consumption that could not satisfy their needs. It may also be that it is known that the countries are low-income countries that don’t satisfy such needs effectively.

In the present study, the likelihood of stunting was 1.7 times higher in children who had no good hygiene practice as compared to children who had good hygiene practice. The study conducted in Dale, Ethiopia [48] was shown to have the same outcome. The possible explanation may be that children who did not keep their hand hygiene and consumed water from unimproved sources have been susceptible to water and fecal-borne diseases. The likelihood of stunting was 1.72 times greater in children who utilized non-improved latrine facilities when compared with children who did have improved latrine facilities. This finding is in agreement with another study conducted in Adwa, Ethiopia [52]. The possible explanation may be that improved latrine facilities can diminish the spread of intestinal worms such that they reduce the status of under-nutrition [53].

Stunting and thinness were 2.74 times and 1.74 times more likely to occur among children who live in food unsecured households, when compared with children who live in food secured households, respectively. Studies conducted in Iran [54] and Durbete, Ethiopia [55] found similar results. This may be due to household food insecurity, which can determine the intake of adequate dietary diversity, and the child could be subjected to poor nutritional status [56]. Children who had no access to treated water were 1.72 times more likely to be thin than children who had access to treated water. This study’s finding are in line with the study findings done in Java, Indonesia [57]. Children who have no access to improved water are 1.76 times more likely to be stunted than their counterparts. This is also in line with the study results conducted in Bangladesh and Iran [36, 37]. This might be due to the fact that water is one of the vehicles for disease transmission, and a history of illness can contribute to the loss of appetite and under nutrition. The likelihood of being thin was 2.67 times higher in children who ate less than 3 meals per day compared to children who consumed more than four. This finding agrees with the outcome of a study conducted in Adawa, Ethiopia [52]. This result may be explained by a low meal frequency per day that could limit the adequate intake of food to maintain the normal nutritional status of the child.

This study shares the limitations of cross-sectional studies and is also restricted to anthropometric measurements and questioning. The study did not measure the micro-nutrient sufficiency of food. Also, it lacks the ability to assess the socio-cultural dimensions like food taboos that can affect the nutritional status of school-aged children. This study provides some insights about the association between under-nutrition and intestinal parasitic infection.

## Conclusion

Stunting and thinness are predominant public health problems in the study area, provided that the prevalence of stunting is slightly higher than that of a national survey on health and nutrition in schoolchildren, whereas the prevalence of thinness is lower when compared to the same national survey [13]. The frontline factors that were significantly associated with stunting in this study were low dietary diversity, poor hygiene practice, a non-improved latrine facility, unsecured household food, and a lack of access to an improved water source. On the other hand, thinness was significantly associated with eating fewer than 3 meals per day, having unsecured household food, and using non-treated water. So, an integrated strategy (government, NGOs, schools, agricultural communities, and advocates) is important to improve the nutritional status of school-aged children, and it is recommended to strengthen nutritional education, promotion of hygiene and sanitation, along with household water treatment and continuing deworming of school children to alleviate under nutrition in the current study area.
